# Comparison of Three Methodologies for Removal of Random‐Noise‐Induced Biases From Second‐Order Statistical Parameters of Lidar and Radar Measurements

**DOI:** 10.1029/2021EA002073

**Published:** 2021-12-30

**Authors:** Jackson Jandreau, Xinzhao Chu

**Affiliations:** ^1^ Cooperative Institute for Research in Environmental Sciences University of Colorado Boulder Boulder CO USA; ^2^ Department of Aerospace Engineering Sciences University of Colorado Boulder Boulder CO USA

**Keywords:** lidar, radar, gravity waves, interleaved method, variance and covariance, potential energy density

## Abstract

Random‐noise‐induced biases are inherent issues to the accurate derivation of second‐order statistical parameters (e.g., variances, fluxes, energy densities, and power spectra) from lidar and radar measurements. We demonstrate here for the first time an altitude‐interleaved method for eliminating such biases, following the original proposals by Gardner and Chu (2020, https://doi.org/10.1364/ao.400375) who demonstrated a time‐interleaved method. Interleaving in altitude bins provides two statistically independent samples over the same time period and nearly the same altitude range, thus enabling the replacement of variances that include the noise‐induced biases with covariances that are intrinsically free of such biases. Comparing the interleaved method with previous variance subtraction (VS) and spectral proportion (SP) methods using gravity wave potential energy density calculated from Antarctic lidar data and from a forward model, this study finds the accuracy and precision of each method differing in various conditions, each with its own strengths and weakness. VS performs well in high‐SNR, yet its accuracy fails at lower‐SNR as it often yields negative values. SP is accurate and precise under high‐SNR, remaining accurate in worse conditions than VS would, yet develops a positive bias under low‐SNR. The interleaved method is accurate in all SNRs but requires a large number of samples to drive random‐noise terms in covariances toward zero and to compensate for the reduced precision due to the splitting of return signals. Therefore, selecting the proper bias removal/elimination method for actual signal and sample conditions is crucial in utilizing lidar/radar data, as neglecting this can conceal trends or overstate atmospheric variability.

## Key Points

- We demonstrate for the first time an altitude‐interleaved method for eliminating random‐noise‐induced biases from second‐order statistics
- The interleaved method is compared to the spectral proportion and variance subtraction methods to reveal strengths and limitations of each
- Lidar data from Antarctica & a forward model are used to assess accuracies and precisions of the three methods under different conditions

## 1 Introduction

Lidar and radar systems provide unrivaled monitoring of the middle and upper atmosphere, allowing for high‐temporal/spatial resolution measurements of atmospheric parameters and constituents, which in turn enable the quantification of complex processes like atmospheric fluxes, constituent transport, turbulence, and gravity waves (e.g., Gardner & Liu, 2010; Hocking, 1996; Lu et al., 2015). These systems can observe a wide range of altitudes by taking advantage of various signal‐return mechanisms (e.g., Baumgarten, 2010; Chu et al., 2020; Chu, Yu, et al., 2011; Kaifler & Kaifler, 2021), which allows detailed studies into vertical coupling, analyzing how atmospheric processes develop as they travel over a wide range of altitudes. Such sophisticated systems have led to decades of impressive remote sensing campaigns (e.g., Li et al., 2020; She et al., 2019; Stober et al., 2021), yet to make full use of the data, advances must be made in data handling. Advances in science inevitably require the use of second‐order statistics such as variances and fluxes, which are inherently biased by random‐noise in the data generated during the detection processes (e.g., Chu et al., 2018; Gardner & Liu, 2014; Whiteway & Carswell, 1995). These biases grow increasingly problematic when analyzing the higher or wider reaches of lidar and radar data. To deal with these biases, various correction methods have been developed over the years (e.g., Chu et al., 2018; Gardner & Chu, 2020; Whiteway & Carswell, 1995), each with its own advantages and disadvantages. These methods have not yet been compared side‐by‐side to assess their effectiveness under various conditions, which are therefore the subject of this work.

This study focuses on lidar‐measured variance and covariance, but the same principles apply when calculating other second‐order statistics using radar and lidar data. Variance is a statistic dependent on the perturbation of a value from its mean, so it is important to understand the anatomy of the perturbation, which can be represented as

(1) $$\begin{array}{l} r_{\text{Total}}^{\prime }\left(z,t\right)=r\left(z,t\right)-r_{0}\left(z\right)=r^{\prime }\left(z,t\right)+\Delta r\left(z,t\right) \end{array}$$

where the stand‐in variable r represents an arbitrary atmospheric parameter (such as density, temperature, zonal, meridional, or vertical wind, etc.) and z and t represent altitude and time, respectively. Here, $r_{\text{Total}}^{\prime }$ is the total measured perturbation that consists of two components: $r^{\prime }$ which is the perturbation caused by atmospheric waves, and Δr which is the noise‐induced perturbation. The $r_{\text{Total}}^{\prime }$ is found by subtracting the mean $r_{0}\left(z\right)=\bar{r\left(z,t\right)}$ from r, where the overbar denotes the sample time average over the chosen observational period. It is worth noting that for some real observations it may be suitable to subtract the sample median, instead of the sample mean, as the median can be more robust than the mean and less biased by outliers.

If calculating the variance of wave‐induced perturbation $\text{Var}\left[r^{\prime }\left(z\right)\right]≃\bar{{\left[r^{\prime }\left(z,t\right)\right]}^{2}}$ using the measured perturbation $r_{\text{Total}}^{\prime }$, then the resultant $\text{Var}\left(r_{\text{Total}}^{\prime }\right)$ would contain both wave‐induced variance and noise‐induced variance, as seen in the right hand side of Equation 2

(2) $$\begin{array}{l} \text{Var}\left[r_{\text{Total}}^{\prime }\left(z\right)\right]=\bar{{\left[r_{\text{Total}}^{\prime }\left(z,t\right)\right]}^{2}}=\bar{{\left(r^{\prime }+\Delta r\right)}^{2}}=\bar{{{\left(r\right)}^{\prime }}^{2}}+\bar{{\left(\Delta r\right)}^{2}}+\bar{2r^{\prime }\Delta r} \end{array}$$

On the righthand side of Equation 2, the second term is the noise‐variance $\text{Var}\left[\Delta r\left(z\right)\right]≃\bar{{\left[\Delta r\left(z,t\right)\right]}^{2}}$ and the third term is the cross‐term between the wave‐induced and noise‐induced perturbations. Because $r^{\prime }$ and Δr are independent and Δr is random and possesses a zero‐mean, the expectation of the cross term $E\left[2r^{\prime }\Delta r\right]=E\left[2r^{\prime }\right]\cdot E\left[\Delta r\right]=0$, that is, this cross‐term will vanish when averaging over a large number of samples. However, the noise‐variance term Var(Δr) will remain and contribute a positive bias. Such a bias must be corrected to accurately estimate the wave‐induced variance. It is important to note that all the overbars above represent the sample average over time, not ensemble average (e.g., Gubner, 2006). Statistics performed in this kind of geophysical studies have assumed that the sampled perturbations induced by atmospheric waves arise from a stationary, ergodic random process. Therefore, the sample average can be used to approximate the ensemble average (Gardner & Chu, 2020), and in this case, the variance computed as the sample average like above can approximate the ensemble expectation of the variance, where “variance” is treated as a signal representing the wave strength, in the limit of very large number of samples. However, the limited number of samples not only differentiates computed variance from the theoretical expectation of signals but also introduces another consequence—the cross‐term may not approach zero enough, adding additional uncertainties to the estimate of wave‐induced variance.

The most direct way to isolate $\bar{{\left(r^{\prime }\right)}^{2}\;}$ is to estimate $\bar{{\left(\mathrm{\Delta r}\right)}^{2}}$ at each altitude and then subtract it from the total variance. As the cross‐term approaches zero due to non‐correlation after averaging over sufficient samples, only the wave‐induced variance will remain. This approach is named the *variance subtraction* method (e.g., Duck et al., 2001; Whiteway & Carswell, 1995). Estimating this term becomes problematic in low‐SNR conditions, as large uncertainties in the estimation often result in the estimated variance‐bias being greater than the geophysical variance itself, yielding a physically‐impossible “negative variance” when the noise term is subtracted.

To overcome this issue, Chu et al. (2018) developed a solution called the *spectral proportion* method, where Monte Carlo simulations based on parameter uncertainties are used to estimate the wave‐occupied proportion p(z) in the total $\;\text{Var}\left(r_{\text{Total}}^{\prime }\right)$ at each altitude. Then the total variance is scaled down to estimate $\text{Var}\left(r^{\prime }\right)=p\left(z\right)\text{Var}\left(r_{\text{Total}}^{\prime }\right)$. By this method, there will be no negative variance induced by the waves. This technique may overestimate $\text{Var}\left(r^{\prime }\right)$ in high‐noise scenarios as the uncertainty in determining p(z) increases substantially.

Gardner and Chu (2020) developed a new approach named the *interleaved* method. In this method, the return photon counts are split into two separate but interleaved samples so that two statistically independent samples probe the same air parcel over the same time period. Consequently, the variance in Equation 2 is substituted with a covariance between these two samples (see Section 2.3 for details) which no longer contains the noise‐induced bias once a statistically‐sufficient sample size is used. This method improves the accuracy of the variance estimate by eliminating the noise‐induced bias yet decreases the precision both through increased uncertainty caused by photon count splitting and by any remaining terms containing the noise‐induced perturbations which have not approached zero under a limited sample size.

Since each approach has strengths and weaknesses, this paper compares these three methods in terms of their accuracy and precision using Antarctic lidar data as well as a forward model. The lidar data used here are the Rayleigh scattering signals collected with an Fe Boltzmann lidar from 2011 to 2020 at the Arrival Heights Observatory near McMurdo Station (Chu, Huang, et al., 2011; Chu et al., 2020; Chu, Yu, et al., 2011). Although these techniques are demonstrated on lidar measurements, they can be applied to radar data as both are similarly affected by noise‐induced biases in higher‐order parameters. Additionally, this paper demonstrates an alternative way to apply the interleaved method by interleaving in altitude as opposed to time‐interleaving as initially demonstrated in Gardner and Chu (2020).

## 2 Three Methodologies

This paper calculates gravity wave potential energy mass density ($E_{\text{pm}}$) to demonstrate these methods, as it is directly proportion to the atmospheric wave‐induced variance discussed in the introduction and it has been studied extensively using lidar. $E_{\text{pm}}$ is calculated as

(3) $$\begin{array}{l} E_{\text{pm}}\left(z\right)=\frac{1}{2}{\left[\frac{g\left(z\right)}{N\left(z\right)}\right]}^{2}\cdot \frac{\text{Var}\left[r^{\prime }\left(z\right)\right]}{r_{\text{Bkg}}^{2}\left(z\right)} \end{array}$$

where g(z) is gravitational acceleration and $r_{\text{Bkg}}\left(z\right)$ is the background value of the parameter. For Equation 3 specifically, r must be either atmospheric temperature or density by the definition of $E_{\text{pm}}.$ N(z) in Equation 3 is the buoyancy frequency and its square is defined as below

(4) $$\begin{array}{l} N^{2}\left(z\right)=-g\left(z\right)\left[\frac{1}{\rho_{Bkg}^{}\left(z\right)}\frac{d\rho_{Bkg}^{}\left(z\right)}{dz}+\frac{g\left(z\right)}{{c_{s}}^{2}\left(z\right)}\right]=\frac{g\left(z\right)}{T_{\text{Bkg}}\left(z\right)}\left[\frac{dT_{\text{Bkg}}\left(z\right)}{dz}+\frac{g\left(z\right)}{c_{p}}\right] \end{array}$$

$N^{2}$ can be calculated either from temperature gradient or from atmospheric density gradient and speed of sound $\;\left(c_{s}=\sqrt{\frac{c_{p}}{c_{v}}R\cdot T_{\text{Bkg}}}\right).$ Here, $T_{\text{Bkg}}\left(z\right)$ is the background temperature, $\rho_{Bkg}^{}\left(z\right)$ is the background density, and $c_{p}$ and $c_{v}$ are the specific heat at constant pressure and constant volume, respectively. We use the density‐based $N^{2}$ calculation for estimating $E_{\text{pm}}$ from density, and the temperature‐based $N^{2}$ to calculate $E_{\text{pm}}$ from temperature, though the two yielded nearly identical results.

In practice, we cannot calculate the $r^{\prime }$ term directly but must estimate it from $r_{\text{Total}}^{\prime }$, meaning that the resulting total $E_{\text{pm}}$ is proportional to $\text{Var}\left(r_{\text{Total}}^{\prime }\right)$, that is, $E_{\text{pm,Total}}\propto \text{Var}\left(r_{\text{Total}}^{\prime }\right)$ which includes the Δr terms:

(5) $$\begin{array}{l} E_{\text{pm},\text{Total}}\left(z\right)=\frac{1}{2}{\left[\frac{g\left(z\right)}{N\left(z\right)}\right]}^{2}\cdot \frac{1}{r_{\text{Bkg}}^{2}\left(z\right)}\left\{\text{Var}\left[r^{\prime }\left(z\right)\right]+\text{Var}\left[\Delta r\left(z\right)\right]+2\cdot \bar{\left[r^{\prime }\left(z,t\right)\cdot \Delta r\left(z,t\right)\right]}\right\}. \end{array}$$

In Equation 5, the last two terms are not wave‐induced but introduced by noise, with the second introducing a positive bias and the third term introducing additional noise. Although $E_{\text{pm}}$ is used as the demonstration here, atmospheric kinetic energy $E_{k}$ has similar issues with noise‐induced bias, as $E_{k}\propto \frac{1}{2}\left[\bar{{\left(u^{\prime }\right)}^{2}}+\bar{{\left(v^{\prime }\right)}^{2}}\right]$ where $u^{\prime }$ and $v^{\prime }$ are the wave‐induced wind perturbations, and therefore can be treated similarly to $E_{\text{pm}}$.

### 2.1 Noise‐Variance Subtraction

As seen in Equation 5, when the $E_{\text{pm}}$ is calculated using $r_{\text{Total}}^{\prime }$, there are noise terms present alongside the wave‐induced $E_{\text{pm}}.$ Even after averaging sufficient samples to drive the third term of Equation 5 to zero, there is still a bias caused by the noise‐induced variance. In response, the noise‐variance subtraction method was introduced, which estimates and subtracts the Var(Δr) term from the total variance. Whiteway and Carswell (1995) derived the following expression for the noise‐variance:

(6) $$\begin{array}{l} {\text{Var}}_{\text{VS}}\left[\Delta r\left(z\right)\right]=\bar{\Delta r{\left(z,t\right)}^{2}}=\frac{1}{k}\sum_{i=1}^{k}{\left[\delta r\left(z,t_{i}\right)\right]}^{2} \end{array}$$

where k is the number of short‐average profiles used within one observational sample, δr is the uncertainty in the parameter r. The variance subtraction‐corrected $E_{\text{pm}}$ is calculated as

(7) $$\begin{array}{l} \begin{array}{l} E_{\text{pm,VS}}\left(z\right)=\frac{1}{2}{\left[\frac{g\left(z\right)}{N\left(z\right)}\right]}^{2}\cdot \frac{\text{Var}\left[r_{\text{Total}}^{\prime }\left(z\right)\right]-{\text{Var}}_{\text{VS}}\left[\Delta r\left(z\right)\right]}{r_{\text{Bkg}}^{2}}. \end{array} \end{array}$$

The limitations of this method become obvious when attempting to use it on noisy data. Here, growing uncertainty in parameter error can cause Var(Δr) to become so large that it exceeds $\text{Var}\left(r_{\text{Total}}^{\prime }\right)$, producing a negative value for $E_{\text{pm,VS}}$, a non‐physical result (see Sections 3 and 4).

### 2.2 Spectral Proportion Method

**Figure 1:**
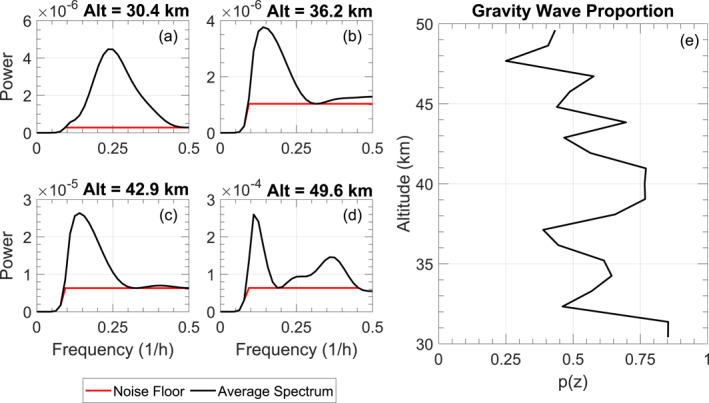
Plots (a–d) demonstrate the spectral proportion method by showing the development of the noise floor at successive altitudes (note the increase in magnitude on the *y*‐axis as altitude increases). Plot (e) shows the altitudinal development of p(z). The data plotted here is from a lidar observation on 21 December 2018.

These negative values led to the development of the spectral proportion method in Chu et al. (2018) which eliminates the possibility of negative $E_{\text{pm}}$. This method also uses the calculated total variance, and then scales it down to estimate the wave‐induced energy. As demonstrated in Chu et al. (2018), we first perform a Monte Carlo simulation by constructing 1,000 copies of r(z,t), adding normally‐distributed noise onto them with a standard deviation equal to the measurement uncertainty at that altitude and time, and calculating the perturbations for each of these 1000‐fields individually. Then, for each altitude, the 1D‐FFT of the perturbations is calculated for each field and averaged over all 1,000 iterations. The noise floor level is then estimated from the averaged‐spectral plots shown in Figures 1a–1d by taking the average of all minima locations above frequency $(f)=0.1\;{\text{hr}}^{-1}$, ignoring the highest minima. We only use minima above $f=0.1\;{\text{hr}}^{-1}$ to avoid the influence of any spectral filters applied during the background subtraction, and we ignore the highest minima to ensure that we do not include any troughs which are actually above the noise floor.

After the noise floor is determined, the proportion of wave energy occupying the total energy p(z) is calculated as

(8) $$p\left(z\right)=\frac{Area\;under\;curve-Area\;under\;noise\;floor}{Area\;under\;curve}$$

Examples of these averaged spectra and noise floors can be seen in Figure 1 for a variety of altitudes while the p(z) is illustrated in Figure 1e. Note that the floor‐level increases with increasing altitude as the corresponding SNR decreases. As a result, p(z) decreases with altitude in general. This p(z) profile is then used to scale down $E_{\text{pm,Total}}$ so that the wave‐induced $E_{\text{pm}}$ is obtained as

(9) $$\begin{array}{l} E_{\text{pm,SP}}\left(z\right)=p\left(z\right)\cdot E_{\text{pm,Total}}\left(z\right) \end{array}$$

This method can, however, introduce positive‐bias under high noise, which is discussed further in Sections 3 and 4.

### 2.3 Interleaved Method

**Figure 2:**
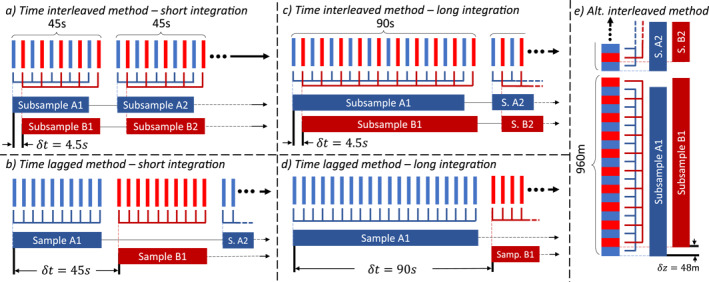
This diagram highlights the advantages of using the time‐interleaved method to calculate covariance by comparing it against a ″time‐lagged″ approach which uses covariance without interleaving the fine bins. (a) and (c) demonstrate that with the interleaved method, the small δt is preserved even if the sample integration time is increased, while (b) and (d) show the large nδt associated with the time‐lagged approach. (e) Shows the altitude‐interleaved concept.

The common idea of the previous two methods was based on the total variance calculation that includes both wave and noise‐induced variances, and then each method employed some algorithm to either remove the estimated noise‐variance or scale down the total variance to estimate the wave‐induced variance. The interleaved method instead eliminates the noise‐induced bias altogether by calculating the covariance of simultaneous, collocated samples taken in a way such that the noise‐terms are driven toward zero. Gardner and Chu (2020) point out that this bias elimination would optimally be done using two adjacent lidars, but such a setup would be complex, expensive, and uncommon. The interleaved method they propose instead introduces a practical way to achieve the same bias‐elimination using a single lidar (see diagrams in Figure 2). Gardner and Chu (2020) have demonstrated interleaving time bins (Figures 2a and 2c) for the covariance calculation but suggested that in many lidar systems it may make more sense to apply it to adjacent altitude bins. Here we describe the altitude‐interleaving method.

Implementation of this altitude‐interleaving process (Figure 2e) is best described by comparing it to a standard lidar data processing approach. In the standard data processing, the photon counts from *n*‐adjacent fine bins, δz in height, are summed into a single, coarse bin with a height of nδz in order to improve the SNR of data. After deriving r from these coarse bins across the entire observation, the data is processed to yield perturbations and variance. In the interleaved method the bins are summed into two separate groups: one a sum of the odd numbered fine bins, and one a sum of the even numbered fine bins. These groupings are then individually processed to generate two distinct sets of atmospheric parameters $r_{A}^{}$ and $r_{B}^{}$ from which perturbations $r_{A,\text{Total}}^{\prime }$ and $r_{B,\text{Total}}^{\prime }$ are derived. Each of these perturbations has the structure of Equation 1, comprised of both wave‐induced and noise‐induced perturbations. We then compute the covariance between these two sets of perturbations as

(10) $$\begin{array}{c} \text{Cov}\left[r_{A,\text{Total}}^{\prime }\left(z_{A}\right),r_{B,\text{Total}}^{\prime }\left(z_{B}\right)\right]\\ =\bar{\left[r_{A}^{\prime }\left(z_{A},t\right)\cdot r_{B}^{\prime }\left(z_{B},t\right)\right]}+\bar{\left[r_{A}^{\prime }\left(z_{A},t\right)\cdot \Delta r_{B}\left(z_{B},t\right)\right]}\\ +\bar{\left[\;r_{B}^{\prime }\left(z_{B},t\right)\cdot \Delta r_{A}\left(z_{A},t\right)\right]}+\bar{\left[\Delta r_{A}\left(z_{A},t\right)\cdot \Delta r_{B}\left(z_{B},t\right)\right]} \end{array}$$

where $z_{A}$ and $z_{B}$ are altitudes representing samples A and B and are separated by vertical distance δz, regardless how large the number n is. It is worth noting that $r_{A}^{\prime }$ and $r_{B}^{\prime }$, the wave‐driven atmospheric perturbations, are highly correlated as they are measured at the same time over approximately the same altitude, shifted by a small value δz. Therefore, their covariance (first term in the righthand side of Equation 10) should be very similar to the variance $\bar{{\left(r^{\prime }\right)}^{2}}.$ In contrast, $\Delta r_{A}^{}$ and $\Delta r_{B}$ are statistically independent random perturbations with zero means so their covariance and terms containing them, that is, the last 3 terms in Equation 10, will approach zero when averaging over a sufficient number of samples. If the number of samples is sufficiently large, all three terms will drop out, leaving a wave term without noise‐induced bias.

The difference between the wave‐induced variance and covariance depends on the level of correlation between $r_{A}^{\prime }$ and $r_{B}^{\prime }.$ If $r_{A}^{\prime }$ and $r_{B}^{\prime }$ are taken simultaneously over the exactly same altitudes, then their covariance is exactly equal to the wave‐induced variance. With the small δz shift in altitude (similar to the small shift in time δt when using the time‐interleaved method), the covariance is slightly smaller than the variance as theoretically derived in Gardner and Chu (2020). However, if the shift gets larger, the covariance will be considerably lower than the variance. Early works which suggested the viability of this covariance‐substitution (Gardner & Liu, 2014) took covariance between samples of alternating coarse‐bins, which is termed “time‐lagged method” here as shown in Figures 2b and 2d, instead of generating two subsamples created at the fine‐bin level as is done in the interleaved method (Figures 2a and 2c). The downside of this time‐lagged approach is that the two coarse‐bins are separated by a large time shift nδt weakening the correlation in the first term of Equation 10 and yielding a falsely‐lower covariance. In the time‐interleaved method, the data is split so that samples A and B are measuring the same parcel of atmosphere over nearly the same time. By minimizing δt as demonstrated in Figures 2a and 2c, the difference between $\text{Var}\left(r_{\text{Total}}^{\prime }\right)$ and $\text{Cov}\left(r_{A,\text{Total}}^{\prime },r_{B,\text{Total}}^{\prime }\right)$ is minimized. That difference can be corrected by a correction factor provided in Gardner and Chu (2020) which is often minimal and can be ignored as long as the interleaved method is used properly. This same theory applies when interleaving in altitude (see Figure 2e). By minimizing δz, the correlation between $\;r_{A}^{\prime }$ and $\;r_{B}^{\prime }$ is maximized, meaning that the covariance can now be substituted into Equation 3 to calculate $E_{\text{pm}}$ free of photon‐noise‐induced bias:

(11) $$E_{\text{pm,INT}}\left(z\right)=\frac{1}{2}{\left[\frac{g\left(z\right)}{N\left(z\right)}\right]}^{2}\cdot \frac{Cov\;(r_{A,\text{Total}}^{\prime },\;r_{B,\text{Total}}^{\prime })}{r_{Bkg}^{2}\left(z\right)}$$

This flexibility in interleaving‐direction allows for the interleaved method to be used on data taken by many different lidar systems. For example, the Na Doppler lidar used as an example in Gardner and Chu (2020) saves its raw data in time intervals of 4.5 s, which means the data can be very finely time‐interleaved, while the Fe Boltzmann lidar used for this study saves its data in time intervals of 1 min, likely too coarse to use a time‐interleaved approach. However, the Fe Boltzmann lidar saves its counts at a high‐vertical resolution of 48 m, allowing the interleaved method to be utilized altitudinally.

Moreover, as E[Δr]=0, interleaving in derived atmospheric parameters (e.g., wind velocities u,v,w) is equivalent to interleaving in raw data (e.g., radar power or lidar photon counts). Therefore, when applying the interleaved method to radar measurements, one may choose to interleave high‐resolution u,v,w in time or altitude domain if interleaving the raw return signals is challenging. By averaging together odd and even (in either time or altitude) wind values, respectively, to create two independent samples, for example, $u_{odd}^{}$ and $u_{even}^{}$, the covariance $\text{Cov}\left(u_{odd}^{},u_{even}^{}\right)$ will be bias‐free and can be used in place of variance. Researchers using radar techniques like coherent detection may be able to cleverly implement this interleaving idea in the raw data (more analogous to the photon count interleaving for lidars).

## 3 Error Analyses

**Figure 3:**
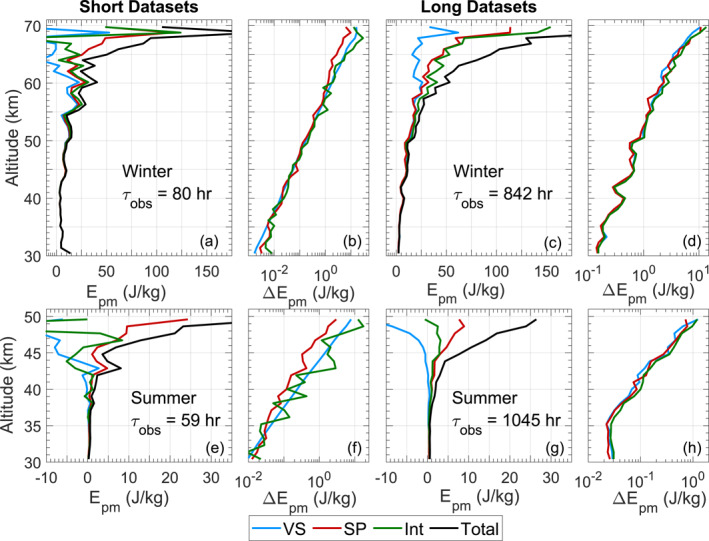
Application of the three bias removal methods to Antarctic lidar data, as well as their uncertainties. Shown here are the $E_{\text{pm}}$ for both large and small samples sizes taken from winter data (July, low noise and high SNRs) in (a–d), and summer data (January, high noise and low SNRs) in (e–h). (b) and (f) show the noise‐induced uncertainties, and (d) and (h) show the total uncertainties, each derived as in Table 1. $E_{\text{pm}}$ is calculated from the atmospheric density perturbations at a binning‐resolution of 1 km and 2 hr, in which the density‐gradient form of Equation 4 is used to derive $N^{2}$ and the speed‐of‐sound $c_{s}$ is calculated from the measured Rayleigh temperature. In the legend, VS: variance subtraction, SP: spectral proportion, INT: interleaved, and Total refers to the uncorrected total $E_{\text{pm}}$.

Results obtained from these three methods are compared in Figure 3, where four cases are shown—winter and summer observations representing high and low SNRs, respectively, with both large and small samples sizes. By comparing these cases, one can get a sense for how the accuracy and precision of each method respond to increased sample size, as well as how they behave in varying noise levels. Before discussing these results in Section 4, it is necessary to introduce the analyses of uncertainty in precision and bias in accuracy.

### 3.1 Precision (Uncertainty) Analysis

The measurement uncertainty (Δr) of parameter r, caused by photon noise and other random noise in lidar detection or by similar random noise in radar detection, introduces uncertainties in the second‐order statistics (such as $\text{Var}\left(r^{\prime }\right)$ and $E_{\text{pm}}$) through the error propagation procedure. If the small errors in $N^{2}$ and $r_{\text{Bkg}}^{2}$ are neglected, the $E_{\text{pm}}$ uncertainty, $\Delta E_{\text{pm}}$, is proportional to $\mathrm{\Delta Var}\left(r^{\prime }\right)$— the uncertainty of $\text{Var}\left(r^{\prime }\right),$ according to Equation 3:

(12) $$\begin{array}{l} \Delta E_{\text{pm}}\;\left(z\right)=\frac{1}{2}{\left[\frac{g\left(z\right)}{N\left(z\right)}\right]}^{2}\cdot \frac{\mathrm{\Delta Var}\left[r^{\prime }\left(z\right)\right]}{r_{\text{Bkg}}^{2}\left(z\right)}. \end{array}$$

We tabulate the uncertainty equations in Table 1, among which Equations (18), (19), (20) consider the photon‐noise‐induced uncertainty only. That is, only the error propagation from Δr is considered while any statistical error (see below) is omitted. The uncertainty in the variance subtraction method is calculated using the noise‐variance given by Equation 6, and the spectral proportion method's uncertainty is found using p(z) as in Chu et al. (2018). In the interleaved method, the uncertainty is increased because of the reduction in SNR caused by splitting the photon counts into two groups. That splitting manifests itself as a $\sqrt{2}$ on δr, thus there is a factor of two in Equation 20. This factor decreases the precision of the measurement, necessitating a larger sample size to reduce the overall uncertainty.

**Table 1:** Root‐Mean‐Square (RMS) Uncertainties of the Estimated Variances and Covariances for McMurdo Observations

Random‐noise‐induced uncertainties^a^	
Variance subtraction^b^	(18) $$\begin{array}{l} \Delta {\text{Var}}_{\text{VS}}\left(r^{\prime }\right)=\sqrt{\frac{\Delta t}{\tau_{\text{obs}}}}{\text{Var}}_{\text{VS}}\left[\Delta r\left(z\right)\right] \end{array}$$
Spectral proportion	(19) $$\begin{array}{l} \Delta {\text{Var}}_{\text{SP}}\left(r^{\prime }\right)=\sqrt{\frac{\Delta t}{\tau_{\text{obs}}}}\text{Var}\left(r_{\text{Total}}^{\prime }\right)\left[1-p\left(z\right)\right] \end{array}$$
Interleaved method	(20) $$\begin{array}{l} \Delta \text{Cov}\left(r^{\prime }\right)=2\sqrt{\frac{\Delta t}{\tau_{\text{obs}}}}\left[\text{Var}\left(r_{\text{Total}}^{\prime }\right)-\text{Cov}\left(r_{A}^{\prime },r_{B}^{\prime }\right)\right] \end{array}$$

Total uncertainties (including both statistical and random errors)	
Variance subtraction	(21) $$\begin{array}{l} \Delta {\text{Var}}_{\text{VS,tot}}\left(z\right)=\sqrt{\begin{array}{l} \frac{2\tau }{\tau_{\text{obs}}}{\text{Var}}_{\text{VS}}^{2}\left(r^{\prime }\right)+\frac{2\Delta t}{\tau_{\text{obs}}}\left[2{\text{Var}}_{\text{VS}}\left(r^{\prime }\right)\Delta {\text{Var}}_{\text{VS}}\left(r^{\prime }\right)+\Delta {\text{Var}}_{\text{VS}}^{2}\left(r^{\prime }\right)\right] \end{array}} \end{array}$$
Spectral proportion	(22) $$\begin{array}{l} \Delta {\text{Var}}_{\text{SP,tot}}\left(z\right)=\sqrt{\begin{array}{l} \frac{2\tau }{\tau_{\text{obs}}}{\text{Var}}_{\text{SP}}^{2}\left(r^{\prime }\right)+\frac{2\Delta t}{\tau_{\text{obs}}}\left[2{\text{Var}}_{\text{SP}}\left(r^{\prime }\right)\Delta {\text{Var}}_{\text{SP}}\left(r^{\prime }\right)+\Delta {\text{Var}}_{\text{SP}}^{2}\left(r^{\prime }\right)\right] \end{array}} \end{array}$$
Interleaved method	(23) $$\begin{array}{l} \Delta {\text{Cov}}_{\text{tot}}\left(z\right)=\sqrt{\begin{array}{l} \frac{2\tau }{\tau_{\text{obs}}}{\text{Cov}}^{2}\left(r_{A}^{\prime },r_{B}^{\prime }\right)+\\ \frac{\Delta t}{\tau_{\text{obs}}}\left[2\text{Cov}\left(r_{A}^{\prime },r_{B}^{\prime }\right)\Delta {\text{Var}}_{\text{INT}}\left(r^{\prime }\right)+\Delta {\text{Var}}_{\text{INT}}^{2}\left(r^{\prime }\right)\right] \end{array}} \end{array}$$

*Note*. τ, the correlation time of temperature or density perturbations (∼1 hr); $\tau_{\text{obs}}$, the total observation time length; Δt, the time resolution of data.

^a^ Variance uncertainties are propagated through Equation 12 to calculate $E_{\text{pm}}$ uncertainties.

^b^ ${\text{Var}}_{\text{VS}}\left[\Delta r\left(z\right)\right]$ is given by Equation 6 in the text.

Equations (18), (19), (20) are suitable for estimating the random‐noise‐induced uncertainty for $E_{\text{pm}}$ of individual measurements. If regarding each individual measurement of $E_{\text{pm}}$ as a sample of the overall stationary ergodic signal, then the statistical error of sampling needs to be taken into account when taking the sample time average. Gardner and Chu (2020) have presented extensive analyses on such overall uncertainties of the estimated sample variances and covariances. Here, we apply their results of $\mathrm{\Delta Var}\left(r^{\prime }\right)$ and tabulate the total $\Delta E_{\text{pm}}$ equations as Equation 21 through Equation 23. The statistical error component, the first term of Equations (21), (22), (23), relates the individually measured atmospheric variances to the presumed stationary signal and expresses uncertainty based on the observational length and the correlation time of the parameter $r^{\prime },$ that is, the number of total independent samples taken during the observations for the sample average. Driving this statistical error toward zero requires a large number of samples, so this statistical error introduces a persistent value to the calculated statistical uncertainty.

### 3.2 Accuracy Analysis With Forward Modeling

While the precision of each method can be assessed from Figure 3 and Table 1, the analysis in Section 3.1 does not give much insight into the accuracies of these methods. To address this issue, a forward model was developed to test their performance. As the modeled wave energy is known, it is possible to assess any potential systematic bias introduced by each of the three methods, in addition to the assessment of their uncertainties in precision.

First, the atmospheric number density and wave‐induced perturbations in this density field are modeled as a background with wave‐induced perturbations:

(13) $$\begin{array}{l} \rho \left(z,t\right)=\rho_{0}\left(z\right)+\rho^{\prime }\left(z,t\right). \end{array}$$

**Figure 4:**
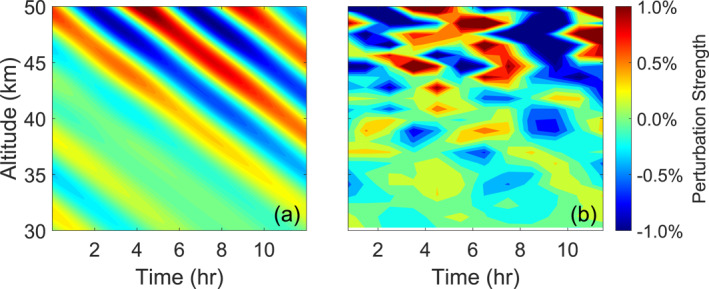
The relative perturbation field generated by the forward model. (a) Shows the simulated perturbation field induced by atmospheric waves, and (b) shows the perturbations derived from the noisy density. This field is the superposition of two plane waves with frequencies and vertical wavenumbers $f=0.182\;{\mathrm{hr}}^{-1}$, $k=0.125\;{\mathrm{km}}^{-1}$ and $f=0.235\;{\mathrm{hr}}^{-1}$, $k=0.160\;{\mathrm{km}}^{-1}$.

The background density field $\rho_{0}^{}\left(z\right)$ is taken from the NRLMSISE‐00 model (Picone et al., 2002). The perturbations $\rho^{\prime }\left(z,t\right)\;$ are modeled as a superposition of two plane‐waves with downward phase progression (as shown in Figure 4a). The wave amplitudes, vertical wave numbers, and frequencies are based on observational data from McMurdo, Antarctica (Zhao et al., 2017). To replicate the growth of gravity wave strength with altitude due to exponentially decreasing background density, the wave amplitudes scale as

(14) $$\begin{array}{l} \frac{\rho^{\prime }\left(z\right)}{\rho^{\prime }\left(z_{0}\right)}=\sqrt{\frac{\rho_{0}^{}\left(z\right)}{\rho_{0}^{}\left(z_{0}\right)}}\; \end{array}$$

where z and $z_{0}$ denote altitude and a reference altitude, respectively.

Next, we simulate the photon return from a lidar shooting vertically into this modeled density field. The photon counts are generated by

(15) $$N_{\text{Wave}}\left(z,t\right)=\left(\frac{z_{0}^{2}}{z^{2}}\right)\cdot N\left(z_{0}\right)\cdot \frac{\rho_{\text{0}}\left(z\right)}{\rho_{\text{0}}\left(z_{0}\right)}\cdot \left[1+\rho_{\text{rel}}^{\prime }\left(z,t\right)\right]$$

where the relative perturbations are defined as

(16) $$\begin{array}{l} \rho_{\text{rel}}^{\prime }\left(z,t\right)\equiv \frac{\rho^{\prime }\left(z,t\right)}{\rho_{0}^{}\left(z\right)}\;. \end{array}$$

Equation 15 is essentially a modification of the general lidar equation for Rayleigh scattering as written in Chu and Papen (2005) with the addition of the density perturbations. $N\left(z_{0}\right)$ is representative of typical photon counts observed by the Fe Boltzmann lidar at a reference altitude (typically around $z_{0}=30\;\text{km}$). After including background photons resulting from the sunlight or star light, we get

(17) $$\begin{array}{l} N_{\text{Total}}\left(z,t\right)=N_{\text{Wave}}\left(z,t\right)+N_{\text{Bkg}}\left(t\right). \end{array}$$

$N_{\text{Bkg}}$ represents the photon counts from these unwanted sources and is estimated by averaging the Fe Boltzmann lidar's photon counts from 150–180 km over many observations during the appropriate season. Poisson‐distributed noise is then added to $N_{\text{Total}}\left(z,t\right)$ by using the photon count at each altitude‐time grid point as the rate parameter for a Poisson‐distributed sampling, resulting in $N_{\text{received}}\left(z,t\right)$, which is a matrix of noisy counts resembling those received by the photon counting system.

**Figure 5:**
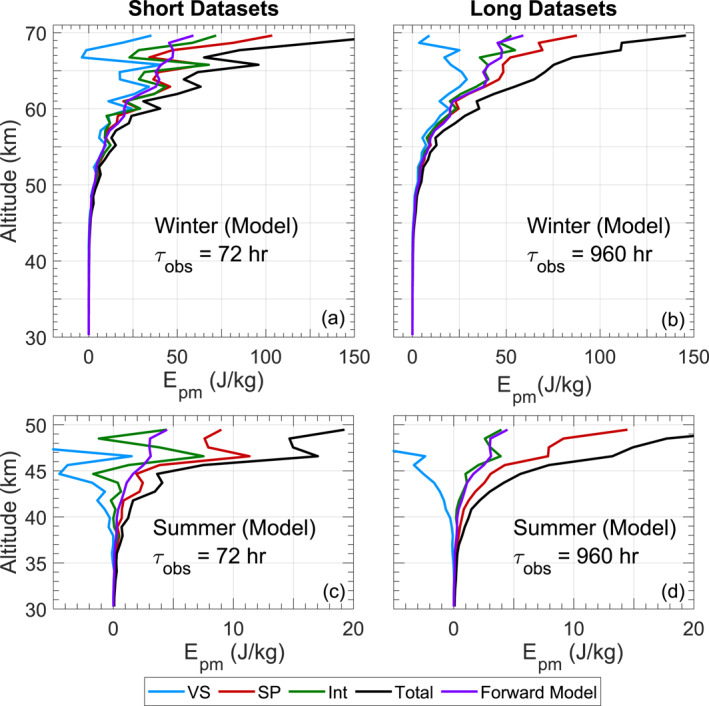
Application of the three bias‐removal/elimination methods to the forward‐modeled lidar density measurements. Density derivation, $E_{\text{pm}}$ calculation, and plotting procedures are identical to those used for Figure 3 except the data used here is from the forward model introduced in Section 3.2. The profile labeled “Forward Model” in each subpanel shows the $E_{\text{pm}}$ as calculated directly from the modeled wave field, which is treated as the reference, that is, a bias‐free profile.

$N_{\text{received}}\left(z,t\right)$ is then treated as if it were a real lidar observation and processed as usual using all three bias‐removal/elimination methods. $N_{\text{Wave}}\left(z,t\right)$ is processed identically to $N_{\text{received}}$ to generate a reference for the true, modeled $E_{\text{pm}}$ in the field with no bias from photon noise. This simulation process was repeated with the same wave spectra many times, each iteration with distinct, random noise, and the results from each method were averaged to create Figure 5. We used the same spectra in generating the wave‐field in each of these plots to satisfy the stationary signal requirement (discussed in Section 1) for averaging large amounts of data, which also serves to minimize any additional effects which could obscure the accuracy of the methods.

## 4 Comparison of Three Methods

We now analyze how the three methods perform under different conditions of SNR and number of samples, in terms of their accuracy and precision. We define accuracy as how close the results are to the true atmospheric $E_{\text{pm}}$ and precision as how well the results are determined (with no reference to the true values) as well as how repeatable the results would be (Bevington & Robinson, 2003). In this dataset, the summer measurements have lower SNRs than winter due to the full sunlight under which the data was collected. Additionally, as this data is derived from Rayleigh scattering, higher altitudes correspond to lower SNRs due to the exponential decrease of atmospheric number density with increasing altitude. Both temperature and density can be derived from these Rayleigh scattering signals. For this study, we use density, but these same $E_{\text{pm}}$ values can also be derived from temperature. This relation is explored further in Appendix A.

We first compare the precision of these methods under various conditions. The precision of each method is largely determined by SNR and is increased with the use of a greater sample size (as the uncertainty is decreased). This is evidenced by Equations (18), (19), (20), (21), (22), (23), as the Δr term (which reflects SNR) scales directly with the uncertainty, and the $\frac{\Delta t}{\tau_{\text{obs}}}$ term scales down this uncertainty as the number of observations increases. In Figures 3a and 3e, the variance subtraction and the interleaved method cannot be scientifically interpreted as shown due to their negative $E_{\text{pm}}$ values. The spectral proportion method appears to show a realistic trend, even when using the small sample size. In Figures 3c and 3g, as more samples are incorporated, the variance removal and spectral proportion method continue to show similar, yet more precise trends. The interleaved method now yields nearly‐entirely positive results which trend at a lower $E_{\text{pm}}$ than the spectral proportion derived results.

We then assess the accuracy of the methods using the forward modeled results in Figure 5. The performance of the variance subtraction method in all conditions clearly shows that this method has low accuracy under high‐noise due to its negative values in Figure 3 and its strong departure from the modeled $E_{\text{pm}}$ seen in Figure 5. While the spectral proportion method yields a precise profile in all cases, Figures 5b and 5d show that it overestimates $E_{\text{pm}}$ when the SNR becomes low, as evidenced by the departure from the modeled $E_{\text{pm}}$. While the interleaved method showed an especially noisy result under a small sample‐size, Figure 5 shows that this noisy profile still generally centers around the modeled $E_{\text{pm}}$, with the same remaining true when using a larger‐sample size.

The negative‐bias of variance subtraction method is due to the uncertainty in the estimation of the noise‐variance. As the noise‐variance is computed using the temperature or density error as in Equation 6, large uncertainties in these error values inevitably occur near the top of the measurement (where the SNR has significantly declined) which cause the noise‐variance to increase dramatically. When subtracted from total variance via Equation 7 these often yield negative variances and thus, physically‐impossible negative $E_{\text{pm}}$ values. Alongside the positive bias caused by the second term of Equation 2, noise can be introduced by the third term if the sample‐size was insufficient to drive it close to zero. While these tests show poor performance from the variance subtraction method at higher‐altitudes, the plots still show that it is a valid approach under high‐SNRs. It has been successfully utilized many times before for lower‐altitude studies (e.g., Chu et al., 2009; Duck et al., 2001; Yamashita et al., 2009).

The positive bias of spectral proportion method seen in Figures 3g and 5d is caused by high‐noise in the initial sample contaminating the spectra at a given altitude. Looking at Figures 1a–1d, there is a regularly occurring peak near f ≈ $0.18\;{\mathrm{hr}}^{-1}$, yet Figure 1d shows additional peaks which are not present at the other altitudes. These could be due to a localized wave present at this altitude throughout the signal, but extensive testing and modeling has revealed that these peaks can be caused by strong noise present in the observation. As this noise‐induced peak disguises itself as a wave‐induced peak, any noise‐floor determination method which captures the energy in the wave‐peaks will also capture the energy in the noise‐induced peak, causing an overestimation in the wave‐energy calculated in the p(z). This systematic underestimation of the noise floor reflects how strong noise is able to cause a positive bias in the form of an overestimated p(z) when using the spectral proportion method, leading to an overestimated $E_{\text{pm}}$ on noisy data.

**Figure 6:**
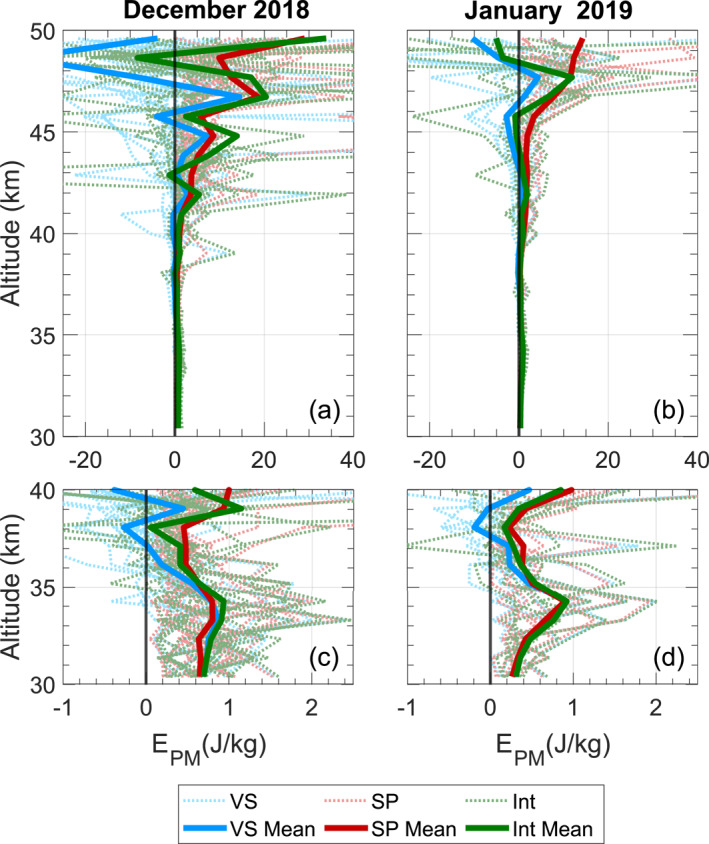
Two monthly $E_{\text{pm}}$ calculations are plotted. The $E_{\text{pm}}$ calculated for an individual run is plotted alongside its monthly mean for both December and January from a single summer season. Additionally, plotted in (c and d) are the lower 10 km for easier comparison.

The interleaved method does not suffer from either positive or negative bias and generally remains centered around the modeled $E_{\text{pm}}$. Its core drawback is the increased uncertainty due to the splitting of the photon counts into two subsamples and additional noise contributed by the final three terms in Equation 10 (if they have not been driven to zero). The bin splitting reduces the signal level in each coarse bin by half, increasing overall uncertainty by $\sqrt{2}$. This increase in uncertainty is significant, and, coupled with the last three terms in Equation 10, can often lead to the derived $E_{\text{pm}}$ displaying negative values. Without enough samples to beat down the uncertainty to a certain level and zero‐out the noise terms from Equation 10, single‐observation results from the interleaved method may not be scientifically meaningful. Additionally, remaining noise‐terms which did not approach zero due to a small sample size will affect the resultant $E_{\text{pm}}$ with the capability to strongly offset the value calculated by the covariance. The stronger the noise‐terms, the more samples are needed to drive the noise terms toward zero and remove their influence. Figure 6 further illustrates these trends by showing the behavior of an entire month of summer observations which is overlayed with its mean. For many of these individual runs, negative values may occur at many altitudes, but given a large enough samples size, the mean value becomes positive. However, even with a large number of samples as shown in Figure 3g, the uppermost bin is still negative, and obviously deviates from the trend established by the bins below. This result reinforces that lower‐SNR increasingly necessitates the use of a larger sample size to compensate for the bin‐splitting‐induced uncertainty increase as well as to facilitate the driving of the noise terms toward zero. Appendix B further elaborates on the development of this precision increase with the increasing sample size.

## 5 Comparison to Previous Results

**Figure 7:**
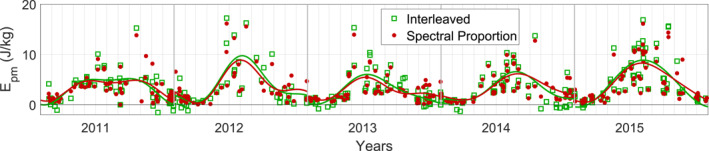
Comparison of year‐to‐year $E_{\text{pm}}$ (altitudinal average from 30–50 km) using the interleaved and the spectral proportion methods. Overlaid are harmonic fittings, calculated as in Equation 19 of Chu et al. (2018) and Equation 4 of Li et al. (2020).

We now compare the performance of the interleaved method to that of the spectral proportion in Figure 7 by replicating results from a previously published study. In Chu et al. (2018), lidar data from 2011 to 2015 taken by the McMurdo Fe Boltzmann lidar was processed with the spectral proportion method to yield $E_{\text{pm}}$. One of the major findings from that study was the strong asymmetry between summer and winter $E_{\text{pm}}$. That study found significantly stronger $E_{\text{pm}}$ in winter as opposed to summer, with this pattern repeating annually.

The comparison in Figure 7 has demonstrated the repeatability of the seasonal asymmetry observations. The interleaved method generally agreed with the trends attained from the spectral proportion method. While some of these interleaved values are negative, it is now known from Section 4 that we must average over many samples (or apply a fit, as we do here) to reveal the true trend. Not only are the observational results replicated by the interleaved method, but since the interleaved method is more accurate in the high‐noise summer and does not overestimate $E_{\text{pm}}$ like the spectral proportion method, the summertime $E_{\text{pm}}$ derived using the interleaved method yields lower summer minimums, and in the winters, a slightly higher maximum. This result means that the major conclusions of Chu et al. (2018) are strengthened by the use of the interleaved method.

## 6 Conclusions and Recommendations

Random‐noise‐induced biases are inherent issues to the accurate derivation of second‐order statistical parameters (such as temperature, wind and species variances, momentum, heat and constituent fluxes, potential and kinetic energy densities of atmospheric waves, and power spectrum estimates) from lidar and radar measurements. As the boundaries of existing research expand, powerful techniques for removal of such biases must be developed to take full advantage of data collection campaigns. The variance subtraction, spectral proportion, and interleaved methods are all viable means to correct for the biases, yet the performance of each method varies depending on the conditions under which they are applied.

Based on the comparisons using the lidar observational datasets from Antarctica as well as the forward‐modeled cases, we draw the following conclusions. The variance subtraction method is best used with high‐SNR observations, as it is easily biased‐negatively by noise in the data. It provides a precise, yet not always accurate, measurement of atmospheric variance even with a relatively‐small sample size. The spectral proportion method is more robust, yielding precise and accurate measurements of variance in significantly noisier data than the variance subtraction method, and also does not rely on a large sample size (Chu et al., 2018). However, it begins to display a positive‐bias under high‐noise conditions. The interleaved method is the only method which will intrinsically not have a bias because it eliminates the random‐noise‐induced biases utilizing two statistically independent datasets that cover the same altitude range and time period (Gardner & Chu, 2020). However, such improved accuracy is attained at the price of reduced precision, necessitating a much‐larger sample size than the others even for high‐SNR measurements.

This work is the first demonstration of altitude/range‐interleaved method for deriving second‐order statistics, following the original proposal by Gardner and Chu (2020). Interleaving in altitude (or range) bins provides two statistically independent samples over the same time period and altitude range even if the original raw data were not saved in high temporal resolutions but sufficiently high spatial (range) resolutions. Therefore, the altitude/range‐interleaved method provides a suitable solution to many current and historic lidar and radar datasets for accurately deriving variances, fluxes, wave energy densities, and power spectrum estimates, etc.

Given the overall considerations we recommend applying the interleaved (either in time or in altitude/range bins) method and the spectral proportion method in real applications because they are superior to the noise subtraction method as demonstrated in this work. When the application goals are to derive statistically mean profiles with high accuracy and/or there are a large number of samples, the interleaved method would be the best choice because it inherently eliminates the noise‐induced biases to give the highest accuracy while the large sample size reduces uncertainties to ensure sufficient precision as well. However, if the application goals are to derive second‐order statistics within a small number of samples and then study the time evolution of such statistics over month, season, and/or year (i.e., non‐stationary signals in longer time periods), the spectral proportion may be a better choice for its higher precision and the ability to handle small sample sizes with a caveat of potentially positive‐biases in high‐noise conditions. Applying the proper bias‐removal method can unlock the full potential of a dataset, allowing retrieval of second or higher‐order parameters into lower‐SNR regions of the data. Additionally, it can reveal trends in the data that may otherwise be concealed by the bias, such as the seasonal asymmetry demonstrated prior, or altitudinal trends which have not yet been discovered.

## Figures and Tables

**Figure A1:**
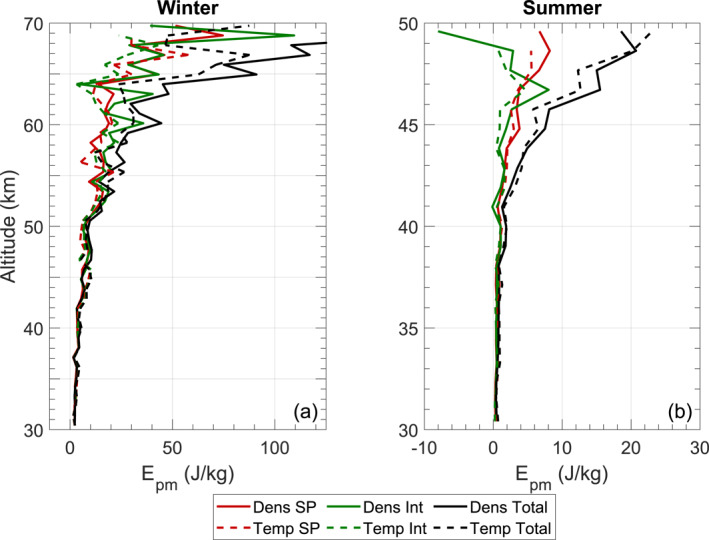
Demonstration of the interleaved and spectral proportion methods applied to $E_{\text{pm}}$ derivations using relative temperature and density perturbations, respectively. Results from the variance subtraction method are not plotted as its values are invalid for much of the range. (a) $E_{\text{pm}}$ profiles from 186 hr of data from July 2019 at McMurdo. (b) $E_{\text{pm}}$ profiles from 180 hr of data from January 2019 and 2020 at McMurdo. The density‐$E_{\text{pm}}$ data here is calculated identically to the other $E_{\text{pm}}$ figures, and the temperature‐$E_{\text{pm}}$ is derived at the same resolution (Δz=1km,Δt=2hr) as the density product.

**Figure B1:**
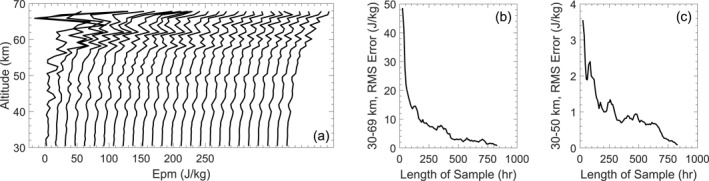
The development of measurement precision with an increasing number of samples, as demonstrated using July lidar observations at McMurdo, Antarctica. (a) The change in $E_{\text{pm}}$ profile as more samples are included in the average, with the leftmost plot showing ∼12 hr of observation, and each successive plot including an additional ∼30 hr. Each profile is shifted by 15 J/kg. (b) The decrease of RMS errors in the altitude range of 30–69 km with an increasing sample length in hours. (c) The decrease of RMS errors in the altitude range of 30–50 km with the increasing sample length in hours.

## Data Availability

The data shown in this work can be downloaded online from https://data.mendeley.com/datasets/5cryh29t67/3.

## Appendix A Considerations on Employing Temperature Versus Density for Rayleigh Lidar

Using Rayleigh lidar data, the gravity wave potential energy mass density $E_{\text{pm}}$ can be calculated from the relative perturbations of atmospheric density or temperature. If done properly, the two will yield the same results. To derive temperatures using the Rayleigh integration technique (Chu et al., 2002) a seed temperature at the top of the altitude range (generally lower SNR) must be used to calculate each point along the altitude profile. The deviation of the empirical seed temperature from the actual value as well as the photon noise at the seeding altitude could result in noisy Rayleigh temperature profiles in the first several scale heights from the seeding point. In contrast, the relative density profile is derived using a bin at the bottom of the altitude range where the SNR is generally higher, reducing the chance of reference‐bin‐noise propagation. More importantly, no seeding temperature is needed for the relative density profiling, thus further removing uncertainties. However, the knowledge of the background temperature profile is still necessary in order to calculate the speed of sound $c_{s}$ for the $E_{\text{pm}}$ computation (see Equations 3 and 4 in Section 2).

While the atmospheric density may seem superior overall in the $E_{\text{pm}}$ derivation, it is often preferred by many authors to use temperature for $E_{\text{pm}}$ studies, as density (e.g., like minor species densities) can reflect high‐rate chemical fluctuations which add additional perturbations to the signal, obscuring the wave dynamics. Fortunately, Rayleigh lidars measure the total atmospheric number density, which is dominated by the chemically stable $N_{2}$ and $O_{2}$; therefore, the Rayleigh lidar‐measured number density does not suffer the chemical fluctuations. Both temperature and density are reasonable approaches for utilizing the Rayleigh lidar data for $E_{\text{pm}}$ studies, as long as the above effects are considered and avoided. It can be helpful to calculate the results via both methods and compare the two, as they should agree if applied properly. This can be seen in Figure A1, where the results are plotted from the Fe Boltzmann/Rayleigh lidar. In this figure, it is clear that even though the uncorrected total $E_{\text{pm}}$ values derived from relative density and temperature perturbations may not agree, the corrected wave‐induced $E_{\text{pm}}$ results derived from both parameters using either the spectral proportion method or the interleaved method generally agree with each other.

## Appendix B Development of Precision With Sample Size in the Interleaved Method

A primary downside of the interleaved method is the reduction of precision caused by splitting the samples into two groups. This is best countered by increasing the number of samples used for the measurements, which reduces the uncertainties roughly by a factor of $\frac{1}{\sqrt{k}}$, where k is the number of independent samples used (Bevington & Robinson, 2003). Figure B1a demonstrates this by showing the development of interleaved profiles with the inclusion of additional samples, where it can clearly be seen that additional samples reduce the noise‐induced uncertainties, particularly those present at high altitudes where the SNRs are low. To quantify the uncertainty reduction, we define the root‐mean‐square (RMS) errors as the difference between the $E_{\text{pm}}$ derived from all samples and the $E_{\text{pm}}$ derived from a smaller sample size. Such RMS errors are similar to the total uncertainties given in Table 1, which include both the statistical errors and the random‐noise‐induced errors. Figures B1b and B1c show that the RMS errors in the altitude ranges of 30–69 km and 30–50 km, respectively, decrease with the increasing sample length in hours at a trend proportional to $\frac{1}{\sqrt{k}}$, with only minor deviations from this trend due to non‐uniform SNRs in individual samples. For our data in July at Antarctica, after 200 hr of data were averaged (i.e., 100, 2‐hr samples), the uncertainty was already decreased by ∼80%. For any observations, the exact number of samples needed to improve the measurement precision depends on the general quality of the actual data, especially its SNRs, as well as the correlation time of the measured atmospheric parameters (see Table 1).
